# Variation in shade-induced flowering in *Arabidopsis thaliana* results from *FLOWERING LOCUS T* allelic variation

**DOI:** 10.1371/journal.pone.0187768

**Published:** 2017-11-08

**Authors:** C. J. Schwartz, Joohyun Lee, Richard Amasino

**Affiliations:** Department of Biochemistry, University of Wisconsin-Madison, Madison, Wisconsin, United States of America; Instituto de Biologia Molecular y Celular de Plantas, SPAIN

## Abstract

Plants have evolved developmental mechanisms to ensure reproduction when in sub-optimal local environments. The shade-avoidance syndrome is one such mechanism that causes plants to elongate and accelerate flowering. Plants sense shade via the decreased red:far-red (R:FR) ratio that occurs in shade. We explored natural variation in flowering behavior caused by a decrease in the R:FR ratio of *Arabidopsis thaliana* accessions. A survey of accessions revealed that most exhibit a vigorous rapid-flowering response in a FR-enriched environment. However, a subset of accessions appeared to be compromised in the accelerated-flowering component of the shade-avoidance response. The genetic basis of the muted response to FR enrichment was studied in three accessions (Fl-1, Hau-0, and Mir-0). For all three accessions, the reduced FR flowering-time effect mapped to the *FLOWERING LOCUS T* (*FT*) region, and the *FT* alleles from these accessions are expressed at a lower level in FR-enriched light compared to alleles from accessions that respond robustly to FR enrichment. In the Mir-0 accession, a second genomic region, which includes *CONSTANTS* (*CO*), also influenced flowering in FR-enriched conditions. We have demonstrated that variation in the degree of precocious flowering in shaded conditions (low R:FR ratio) results from allelic variation at *FT*.

## Introduction

Plants have evolved the ability to alter morphology and the timing of flowering as an adaptation to changes in the local environment. One such mechanism is the shade-avoidance response, which allows plants to compete with neighboring vegetation for sunlight. When challenged with a shaded environment, plant stems and petioles elongate in an attempt to grow above the canopy and capture more light. Moreover, shaded plants often reproduce earlier than non-shaded plants to ensure that at least some limited seed set occurs in the suboptimal environment (for review see [1, 2]).

Changes in light quality are perceived by the photoreceptors including PHYTOCHROMES A-E (PHYA-E), CRYPTOCHROMES 1 and 2, and PHOTOTROPINS 1 and 2 (for review see [3]). In shade, the ratio of red (R) to far-red (FR) light is decreased because neighboring foliage absorbs R light, but reflects FR light. PHYB is the primary phytochrome that elicits a response to decreasing R:FR ratio, with PHYD and PHYE having mostly redundant roles with PHYB in light perception [4]. Natural variation in PHYB can result in different responses to changes in the ratio of R:FR light, providing an avenue for local adaptation [5]. The active form of PHYB elicits a response to changes in light quality by entering the nucleus and affecting transcription of a number of genes [6].

The circadian clock and phytochromes are part of the system that monitors day-length and mediates changes in the expression of gene networks. The circadian clock regulates *GIGANTEA* expression, and *GIGANTEA* is required for *CONSTANS (CO)* expression [7]. *CO* expression oscillates during a 24 hour period, and stability of the CO protein is light dependent, with degradation occurring in the dark [8]. *CO* promotes flowering by activating expression of downstream floral integration genes, such as *FT* and *SUPPRESSOR OF CONSTANS 1*, and *FT* expression is a key inducer of flowering [9]. Thus, both light duration and quality, perceived by phytochromes, influence the timing of reproductive development.

*FT* was initially genetically identified by Koornneef et al. [10] as a delayed-flowering mutant. The *FT* gene, more recently called *FLOWERING LOCUS T*, was cloned in 1999 (11). *FT* is activated by *CO* in long-day photoperiods in long-day plants, such as Arabidopsis [11]. *FT* is now recognized as “florigen,” a mobile signal that promotes flowering (for review see [12]). The ancient *CO/FT* module is highly conserved among plant species, and facilitates several developmental responses, such as the onset of dormancy, in response to changes in photoperiod, an annually consistent environmental cue [13–15].

The control of *FT* expression is an integration point at which multiple environmental signals allow plants to fine tune flowering time as a function of environmental conditions. In addition to photoperiod, *FT* expression is influenced by vernalization (extended cold), ambient growth temperature, and hormone levels [16–20]. Recent work has demonstrated conserved regions within the *FT* promoter that are likely to be key determinants for expression via binding of different proteins or protein complexes [21, 22]. *FLC (FLOWERING LOCUS C)*, a potent floral repressor silenced by vernalization, as well as the repressor SHORT VEGETATIVE PHASE, bind directly to the *FT* promoter [23, 24]. *CO* and GA (gibberellic acid) responsive proteins activate expression of *FT* [9, 25]. If there is allelic variation at *FT* it is likely to be a result of cis-regulatory element variation, because the *FT* amino acid sequence is quite highly conserved among Arabidopsis accessions that are highly variable for flowering time.

In this study, we evaluate natural variation for flowering time in FR-enriched conditions that mimic shade. The majority of Arabidopsis accessions flower rapidly in these conditions, with the exception of two groups of accessions, which appear to be “blind” to the increased FR light. Previous work has demonstrated that FR enrichment overrides the repressive effect of FLC and alters the timing of expression of *GI* and *CO* [26–29]. Here we find FR responsiveness maps to *FT* and is correlated with *FT* expression levels. For one group, *FT* is the only locus identified as causing FR insensitivity, whereas for a second group, both *FT* and *CO* are candidate genes.

## Methods and materials

### Plant material

Accessions were obtained from Julian Maloof and Todd Michaels in 2002. Strains were described as follows: ColFRI [30], *co-9* [31], *ft-3* [10], Est-1 NIL [32].

### Growth conditions

All growth-chamber experiments were conducted in reach-in chambers (Percival Scientific I-60LX) fitted with T12 fluorescent bulbs (Philips Lighting; R:FR approximately 5, PPFD approximately 60–70 umol m^-2^s^-1^) set for long days (LD; 16h of light/8h of dark) or short days (SD; 8h of light/16h of dark). All experiments were conducted at 22C, except for the cycling experiments in which the 16h light period was at 22C and the 8h night period was at 16C. Certain chamber shelves also contained arrays of FR LEDs (Plasma Ireland; wavelengths approximately 735–740 nm) that lowered the R:FR ratio from 5 to 0.15 (low FR) or 0.04 (high FR). Vernalization treatment was done in soil in a coldroom in the dark at 4C. Plants grown outdoors were first grown in growth chambers for 3 weeks and then moved outside to a partially shaded (70%) location in Madison WI beginning in early June. All light measurements were made with a wideband portable spectroradiometer (International Light; RPS900-R).

Plants were phenotyped by counting Rosette Leaf Numbers (RLN) on the primary shoot prior to flowering, with the exception of plants grown outdoors where Days to Flowering (DTF) was used due to excessive growth of secondary shoots. Genotypes were determined by SSLP or CAPS markers using TaKaRa ExTaq RR001A.

### Statistics and QTL mapping

Correlation coefficients were calculated using Microsoft Excel (CORREL). QTL mapping utilized R/qtl (http://www.rqtl.org) in the statistical package R (https://www.r-project.org).

### Primers, markers, and strain polymorphisms

See S6 Table.

### *FT* expression

Plants were genotyped after one week of growth in LD conditions. Plants heterozygous for *FT* were grown for 4 weeks in LD and then shifted to LD + FR or kept in LD conditions.

To monitor *FT* expression with and without FR enrichment, we carried out Semi-quantitative RT-PCR of allele-specific transcripts. Total RNA was isolated from each accession, and cDNA was generated [33]. We used FT specific primers, 5’-CTCAGGAACTTCTATACTTTGGTTATG-3’ and 5’- CTGACAATTGTAGAAAACTGCG-3’ to amplify FT via 28 PCR cycles. The loading control of each reaction was carried out using UBQ10 primers, 5’-CTACCGTGATCAAGATGCAGATC-3’ and 5’-TTGTCGATGGTGTCGGAGCTTTC-3’. To check allele-specific expression, we monitored *FT* expression and its digestion pattern in various heterozygous accessions. To check *FT* expression in Col FRI/Fl-1, we amplified a region of *FT* using a polymorphism between the two accessions (BsmBI does not cut-Col FRI, BsmBI cuts Fl-1). Similarily, FT expression in the other heterozygous accessions was monitored and digested with Cac8I (no Cac8I site: Fl-0 and Mir-0, Cac8I cuts Hau-0 and Col FRI) using primer set (5’-CAATCAACACAGAGAAACCACCTG-3’ and 5’- CTCGCGAGTGTTGAAGTTCTG -3’).

## Results

### Surveying natural accessions for FR flowering responsiveness

**Fig 1:**
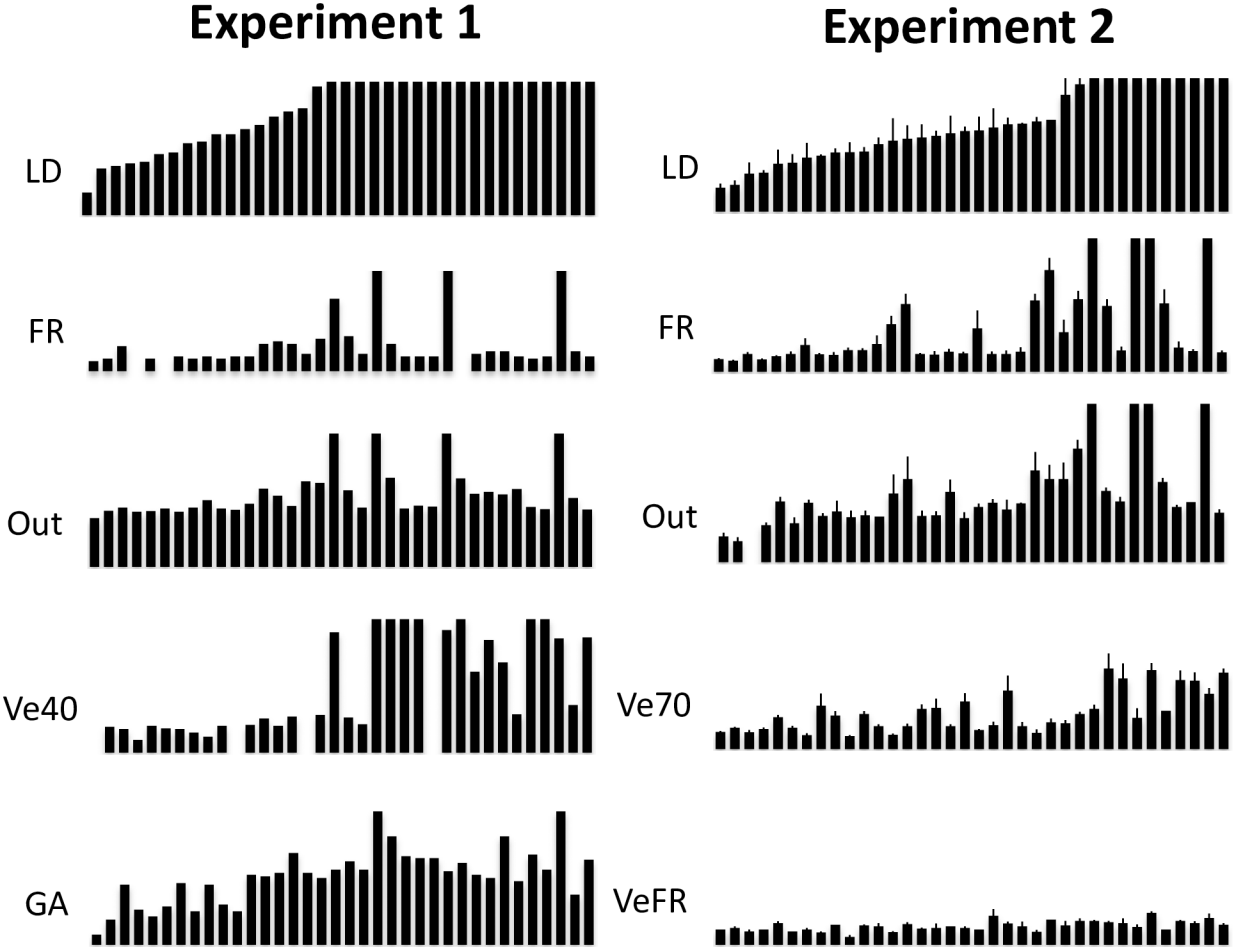
Flowering phenotypes of Arabidopsis accessions under various conditions. The x-axis shows Arabidopsis accessions (S1 Table) sorted according to flowering time in LD. Experiment 1. Initial screen for flowering behavior using one or two plants per accession. FR was on for 24hrs, white light was on for 16hrs. Outdoors experiments were conducted in a partially (70%) shaded plot. All the y-axes are from 0-80RLN, except for FR, which is from 0-20RLN. Experiment 2. Secondary screen for flowering behavior. FR enrichment was only during the light period (16hrs). All axes are 0-80RLN, except for FR, which is from 0-20RLN. 3 plants were evaluated for each accession. Error bars represent standard deviation. Legend; LD (long days), FR (far-red enriched conditions), Out (outside experiment), Ve40 (40 day vernalization), Ve70 (70 vernalization, GA (gibberellic acid treatment), and VeFR (70 day vernalization + far-red enriched conditions).

Our studies of late-flowering, vernalization-insensitive accessions revealed that most of these accessions flower relatively rapidly when exposed to FR-enriched light. However, a small subset does not exhibit accelerated flowering with FR enrichment. To characterize the FR-flowering phenotype and investigate how it relates to other known pathways that accelerate flowering, we grew 36 accessions under 5 conditions, including FR enrichment. The accessions (S1 Table; Experiment 1), mostly winter annuals, were biased towards those that respond poorly to a standard vernalization treatment (40 days at 4°C). As expected many accessions did not respond with greatly accelerated flowering after vernalization, but only a small subset of the accessions did not respond to FR-enrichment with accelerated flowering (Fig 1; Experiment 1). This experiment demonstrates that the vernalization and FR response were distinct in promoting flowering (r = 0.406), and thus may be acting through different floral-promoting pathways. Likewise, treatment with GA was relatively loosely correlated with the FR flowering phenotype (r = 0.571). However, the FR phenotype did correlate well with flowering time recorded outside at a site shaded by foliage approximately 70% of the day (r = 0.878; Fig 1).

To further characterize the FR phenotype, we characterized another set of 36 accessions (Fig 1; Experiment 2). Eight accessions in this set were vernalization insensitive, flowering with more than 30 rosette leaves even after an extended cold exposure (70 days at 4°C). The vernalization phenotype was poorly correlated with the FR phenotype (Fig 1; Experiment 2 (r = 0.19)). However, once again the FR phenotype correlated well with outdoor flowering time (r = 0.941). Interestingly, combining vernalization and FR treatments caused all accession to flower with fewer than 20 rosette leaves (average = 11.2 leaves), demonstrating a synergistic relationship between the two conditions, consistent with FR and vernalization acting through separate floral initiation pathways.

**Fig 2:**
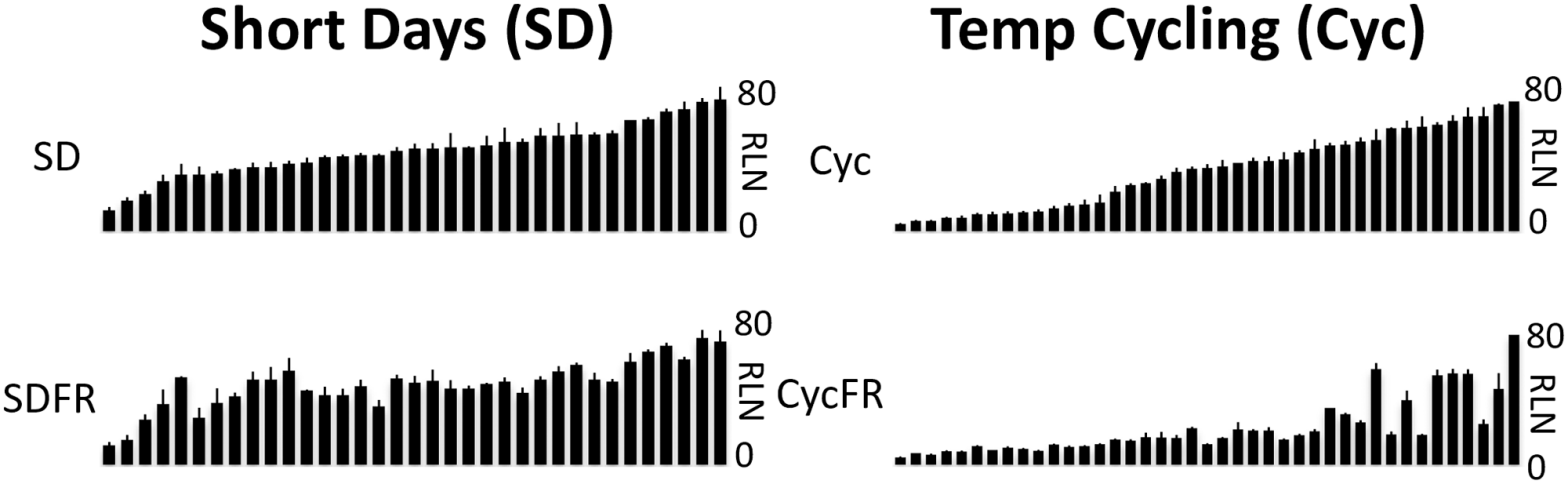
Flowering phenotypes of Arabidopsis accessions in SD and with temperature cycling. The x-axis shows Arabidopsis accessions sorted according to SD or Cyc flowering time. The FR effect on flowering is negligible under SD growth conditions. Temperature cycling does not substitute for, nor curtail FR induced flowering. Y-axes are 0-80RLN.

To assay the effect of photoperiod in promoting flowering, we characterized another set of 36 accessions (S1 Table; SD), biased for spring annuals, in short days (SD) +/- FR (Fig 2). The addition of FR had no significant effect on flowering time (SD average = 37.07 RLN (Rosette Leaf Number); SD + FR average = 35.74 RLN), demonstrating that the FR response is acting through the photoperiod pathway, which is inactive in SD conditions. We also assayed a set of 43 accessions (S1 Table; Cyc) for flowering time under realistic light (22C) and dark (14C) temperature cycling conditions +/- FR. Most accessions flowered earlier with the addition of FR, demonstrating that temperature cycling did not eliminate or enhance the FR effect, suggesting that the two treatments operate through separate pathways, and that the cycling temperature regime we used does not substitute for FR (Fig 2). In fact, temperature cycling had little effect on flowering time in this experiment as demonstrated by the high correlation coefficient in LD and LD + cycling (r = 0.931).

All the above experiments indicate that the FR effect on flowering is dependent on the photoperiod pathway, and the high correlation with flowering time recorded in a partially shaded site outdoors demonstrates that the FR flowering phenotype we observed is not a laboratory anomaly, but is likely to be ecologically relevant.

### Physiology and genetics of FR responsiveness

**Fig 3:**
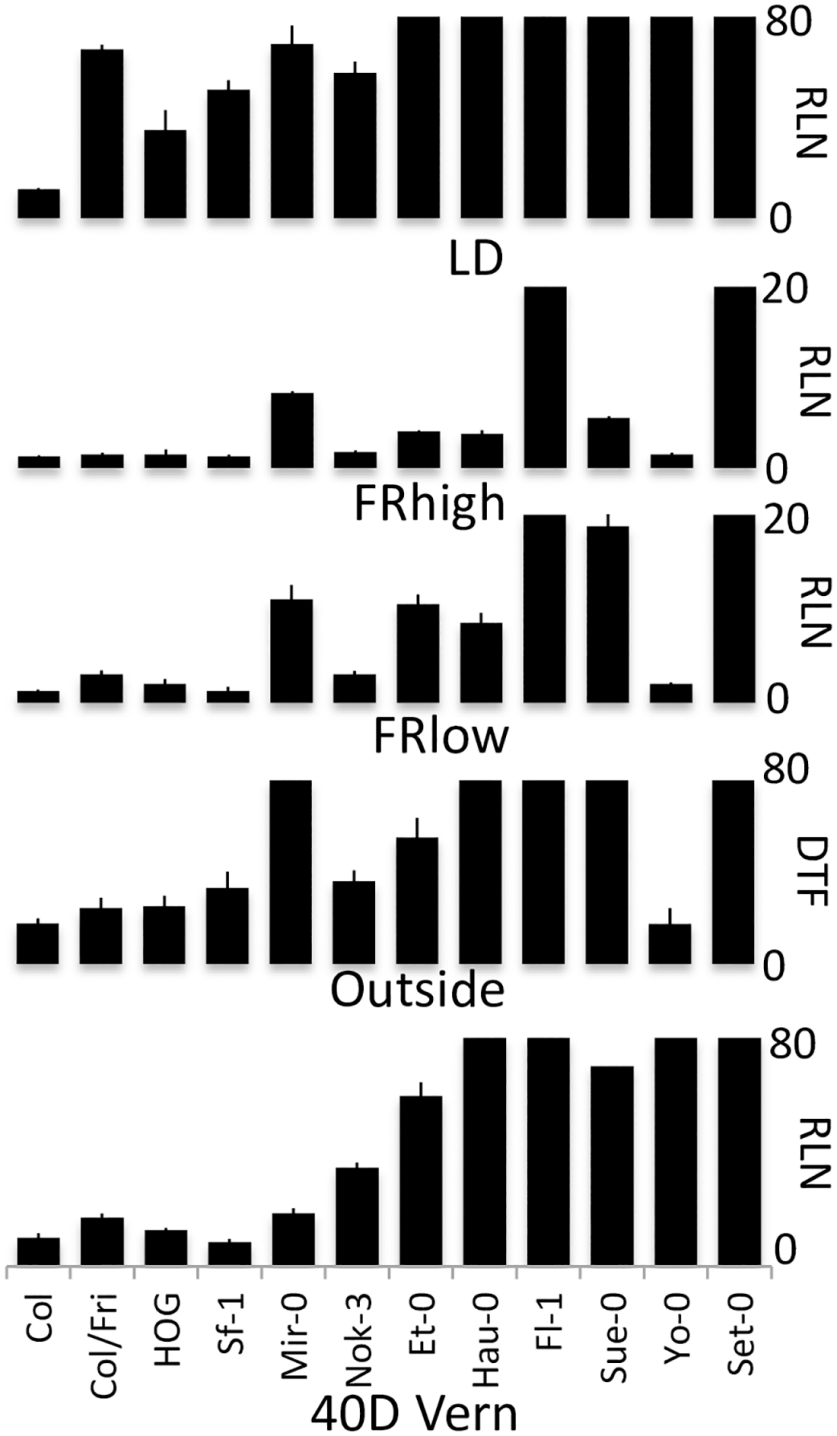
Flowering behavior of selected accessions. Ten accessions, plus controls (Col and ColFRI), grown under 5 different conditions that influence the timing of flowering. The accessions were chosen as representatives of different classes identified in this study. High intensity FR compared to relatively lower intensity FR results in contradictory phenotypes for some accessions, like Sue-0 and Et-0 (FR semi-sensitive accessions).

To further explore the genetic and molecular components underlying the FR effect on flowering, 10 accessions representing much of the flowering-time variation observed in the initial survey experiments, plus the laboratory control strains (Col and ColFRI), were chosen for further study and grown in 10 different conditions (S2 Table). Fig 3 shows the phenotypes of the accessions in the 5 most relevant growth conditions, and correlations between all conditions are shown in S3 Table. As found in the previously described experiments, the flowering phenotypes in FR-enriched conditions were poorly correlated with flowering time after vernalization or with exogenous GA application, and GA largely eliminates the photoperiod effect on flowering in these accessions (S1 Fig). We tested two different intensities of FR, high (R:FR ratio = 0.04) and low (R:FR = 0.16). Some accessions responded robustly to high intensity FR, but not to low intensity FR. Based upon the phenotypes, we were able to group the 10 accessions, plus the laboratory controls Col and ColFRI, into 5 groups (Table 1). Accessions were either vernalization sensitive or insensitive, while FR response fell into three categories, sensitive, semi-sensitive, and insensitive.

**Table 1:** The twelve representative accessions/strains exhibit distinct flowering-time behaviors separable into 5 classes.

Accession	Vern	FR	Class
**Set-0**	insensitive	insensitive	1
**Fl-1**	insensitive	insensitive	1
**Hau-0**	insensitive	Semi-sensitive	1
**Mir-0**	Sensitive	insensitive	2
**Sf-1**	Sensitive	Sensitive	3
**Nok-3**	Sensitive	Sensitive	3
**Col**	Sensitive	Sensitive	3
**ColFRI**	Sensitive	Sensitive	3
**HOG**	Sensitive	Sensitive	3
**Yo-0**	insensitive	Sensitive	4
**Sue-0**	insensitive	Semi-sensitive	5
**Et-0**	insensitive	Semi-sensitive	5

Ten accessions, plus the laboratory strains Col and ColFRI, were grouped into five classes according to vernalization and FR responsiveness. FR and vernalization insensitive (Inv), FR insensitive and vernalization sensitive (InV), FR and vernalization sensitive (SeV), FR sensitive and vernalization insensitive (Sev), and semi-sensitive to FR, but insensitive to vernalization (SSv). Mir-0 and Yo-0 are the sole representatives of their class, and Yo-0 is the only USA accession.

### Characterization and mapping of FR sensitivity in segregating populations

**Fig 4:**
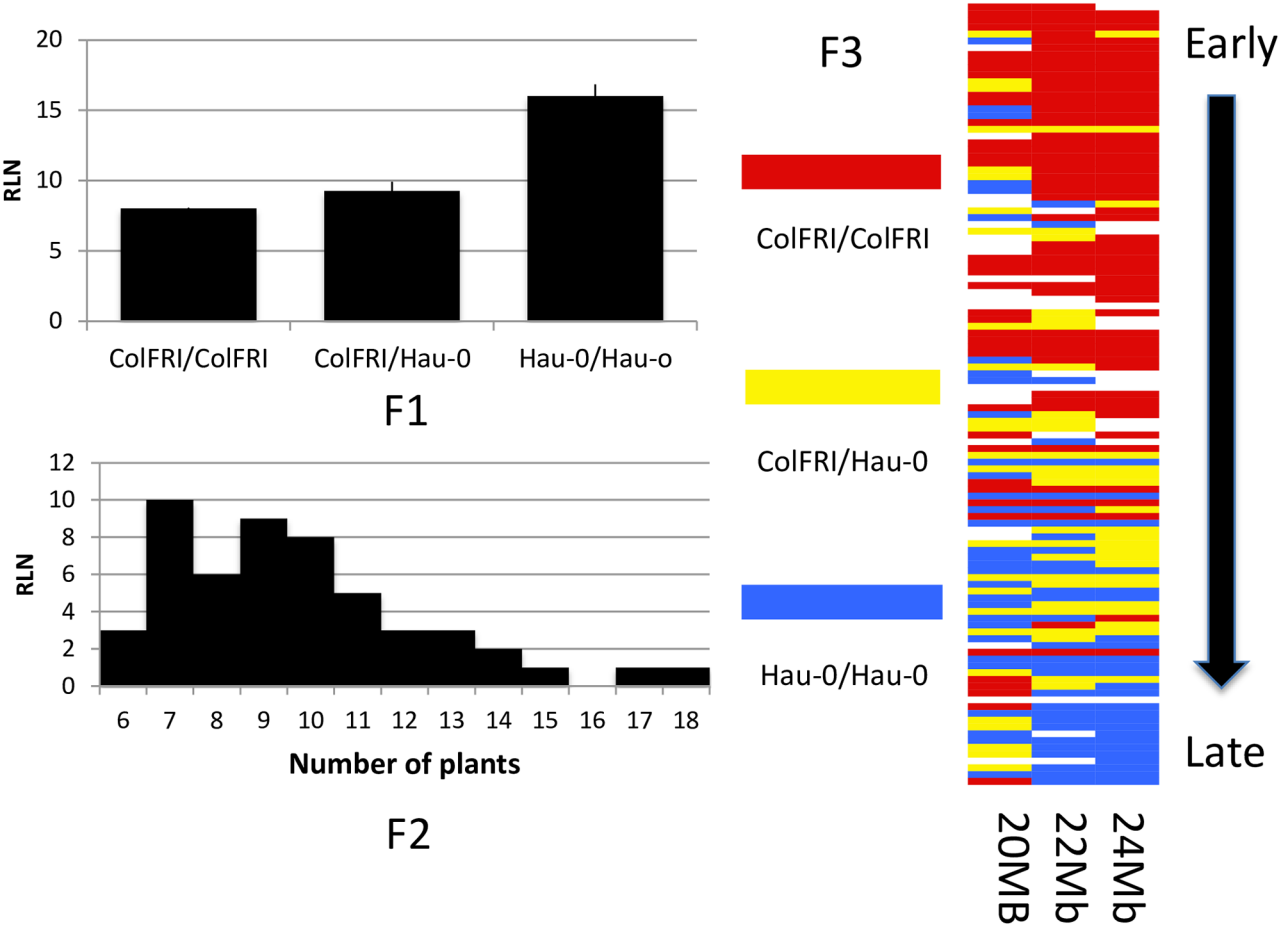
F2, and F3 FR induced flowering phenotypes of the Hau-0xColFRI population, and linkage to the *FT* region (24Mb). Hau-0/ColFRI F1 plants are responsive to FR enrichment similar to ColFRI; thus the causative allele(s) are dominant. The Hau/ColFRI F2 population segregates in a 3:1 manner, suggesting a single large effect locus. Genotyping and phenotyping of a Hau/ColFRI F3 population maps the phenotype to the *FT* region (24Mb).

To determine the genetic basis of the FR-flowering time differences, crosses were performed among the 10 accessions and controls with different FR-flowering behaviors. F1 plants grown +/- FR show that FR responsiveness is largely dominant with a small dosage dependency effect since most F1 plants have a flowering time nearer the early/FR sensitive parent (S2 Fig). Fig 4 shows flowering time data for the F1, F2, and F3 generations derived from a cross off Hau-0 and ColFRI. Hau-0 is semi-sensitive to FR-enhanced flowering, and flowers earlier with FR enrichment, but still much later than the ColFRI control. The semi-sensitivity of Hau-0 greatly facilitated additional genetic analysis, since it will flower in FR conditions, unlike insensitive accessions. In the F1, FR sensitivity was dominant with the phenotype being equivalent to ColFRI. The F2 segregated in a 3:1 manner, suggesting one major effect locus. Rough mapping of the F3 population identified a region (24Mb) of chromosome 1 that was linked to FR sensitivity. To fine map the FR-sensitivity QTL, 700 F2 plants were phenotyped and genotyped. The QTL was reduced to a region of 89.4kb on chromosome 1 that contained the floral integrator gene, *FT*.

Further evidence for the role of *FT* in FR sensitivity was provided by a second population, generated by crossing a flowering time FR-insensitive accession (Fl-1) to ColFRI, in which the FR phenotype in the F2 generation segregated in 3:1 manner with early flowering being dominant (S3 Fig). Twenty four F2 plants were genotyped at a marker near *FT* (F5I14). The results demonstrate the strong effect of the Fl-1 *FT* region in delaying flowering under FR enrichment (ColFRI/ColFRI = 28.5 RLN; Fl-1/Fl-1 = 78 RLN). In addition, *FLC* was genotyped in this population and showed no correlation to flowering time (data not shown).

Evaluation of F2 populations derived from crosses between FR semi-sensitive and insensitive strains to ColFRI demonstrated tight linkage of FR sensitivity to the region of chromosome 1 containing *FT*. For some crosses, multiple independent F2 populations were analyzed, and the results were reproducible (data not shown). As already discussed, the association of the *FT* region and FR-enriched flowering in the Hau/ColFRI and the Fl-1/ColFRI F2 populations is strongly supported, whereas the *FLC/CO* region shows no support. In addition, for crosses between Sue-0 (semi-sensitive) and Et-0 (semi-sensitive) to ColFRI, flowering time was linked in the F2 to the *FT* region (p = 0.001, p = 0.074, respectively). However, one population, Mir-0/ColFRI, has relatively lower support at the *FT* region and significant support for the *FLC/CO* region in comparison to the other F2 populations, indicating natural variability in which locus/loci confers FR sensitivity (this population is discussed in more detail below). In addition, an F2 population from a FR insensitive accession (Mir-0) and a FR semi-sensitive accession (Sue-0) had significant linkage between FR-enhanced flowering and the *FT* region (p = 0.02), as would be expected. Likewise, when Fl-1 (insensitive) was crossed to Hau-0 (semi-sensitive), the F2 FR flowering phenotype was dependent on the *FT* region (p = 0.01).

We utilized a whole-genome scan [34] (149 SNP markers) to evaluate 24 plants from two populations grown outdoors. Previously FR flowering time in an F2 population from Fl-1/ColFRI showed linkage to the *FT* region, and QTL mapping revealed a QTL on chromosome one over the *FT* region LOD = 5.8, LOD >4.2 are significant). For the second F2 population, Mir-0/ColFRI, both chromosome 1 (ColFRI/ColFRI = **35.5** +/- 7.14 DTF; Mir-0/Mir-0 = **54.8** +/- 6.90 DTF) and chromosome 5 (ColFRI/ColFRI = **41.6** +/- 8.82 DTF; Mir-0/Mir-0 = **53.7** +/- 2.25 DTF) had significant support for having an effect on flowering time.

### The role of *CO* in FR sensitivity

The Mir-0 accession displays some unique behavior compared to the other FR-insensitive accessions. First, it is not extremely late flowering without FR (50 RLN), and flowers with approximately the same number of leaves as with or without FR enrichment when grown side by side. Second, Mir-0 is extra sensitive to vernalization, with even short cold treatments causing a reduction in flowering time. To pursue in more detail the behavior of this accession, we grew 96 F2 plants of Mir-0/ColFRI in FR-enriched conditions. The earliest and latest 10 plants were genotyped at a tightly linked *FT* marker (F5I14). The earliest plants were enriched for ColFRI alleles (17/20) and the latest plants were enriched for Mir-0 alleles (15/20). Heterzygous plants were present in both the earliest and latest classes, suggestive of an additional causative locus. Using progeny lines fixed for *FT* (ColFRI) and *FLC* (ColFRI), but heterozygous for *CO* we observed a differential response to FR flowering attributable to the *CO* region (p-value <0.0001). Thus, the almost complete FR insensitivity of Mir-0 is likely to be caused by natural variation at both *FT* and *CO*. A second accession, Br-0, was also different from the other FR insensitive accession, and displayed identical flowering behavior as Mir-0. Both accessions are also glabrous, suggesting possible relatedness. Thus Br-0 was included in the *co* and *ft* mutant analysis described below.

### FR sensitivity is decreased in *FT* and *CO* reduced-function alleles

**Fig 5:**
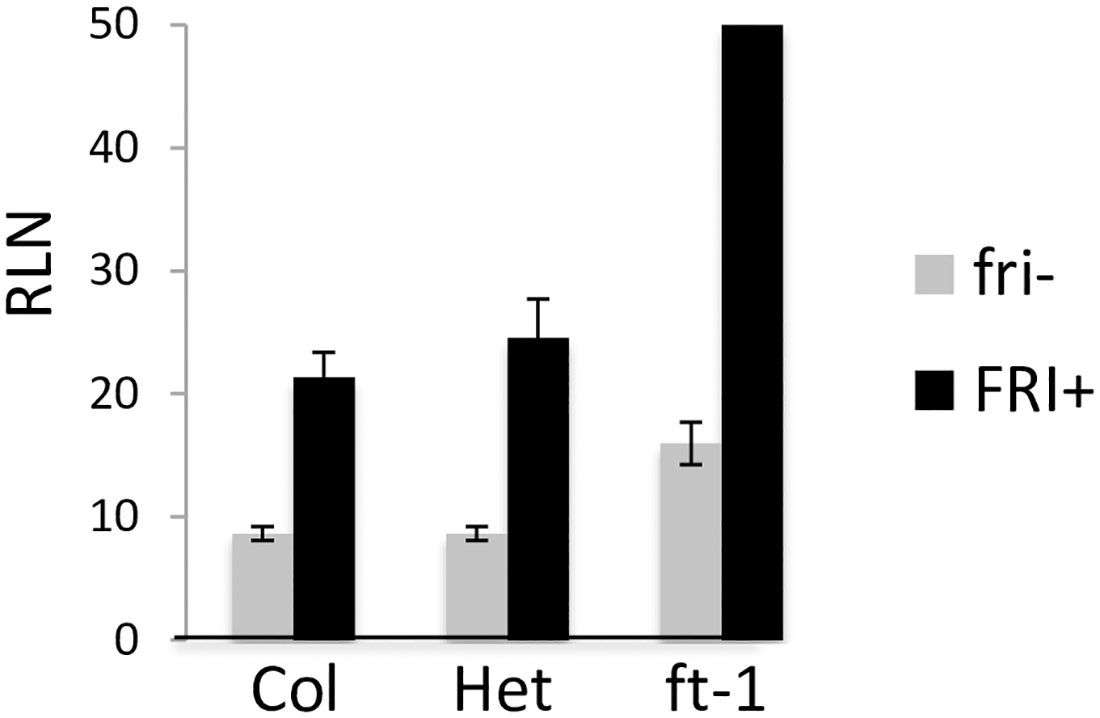
Two loci, *FRI* and *FT*, are sufficient to confer FR insensitivity. An *FT* mutant in Col was integrated into the ColFRI background, to demonstrate the role of *FT* in a late-flowering strain. In the ColFRI background, *FT* is necessary for FR sensitivity.

Given the compelling data for a role of *FT* in FR sensitivity, we evaluated flowering time in an F2 population from a Col *ft* mutant crossed to ColFRI (Fig 5), and the results clearly demonstrate that *FT* is necessary for accelerated flowering in FR-enriched conditions. In plants containing one or two copies of the *FRI* gene, homozygous *ft* plants did not flower in this experiment (80+ RLN), whereas one functional copy of *FT* was sufficient for FR sensitivity and relatively early flowering (<28 RLN). We also evaluated the *co* mutant in the ColFRI background and it also flowered extremely late in FR-enriched conditions, nearly identical to the *ft* mutant in the same genetic background (data not shown).

To further our understanding of the role of *FT* and *CO* in FR responsiveness, we crossed multiple accessions to *ft* or *co* mutants in the ColFRI background and phenotyped the F1 plants for FR sensitivity (S5 Table). It is important to note that interpretation of results for flowering time from these F1 populations can be complicated by the introduction of “functional” dominant *FT* and/or semi-dominant *CO* alleles. Mir-0 and Br-0 when crossed to ColFRI(*ft-1*) flowered substantially later (20+ RLN) than the parental accession, demonstrating an inability of the Mir-0 and Br-0 *FT* alleles to complement the *FT* mutant. In addition, when Br-0 and Mir-0 were crossed to a *CO* mutant, the F1 plants were equally late, comparable to the crosses with *ft*, showing a lack of *co* complementation. The mutant analysis combined with the previously described experiments support the idea that Mir-0 and Br-0 likely have alleles of *CO* and *FT* which do not respond to FR enrichment.

For the majority of accessions analyzed, *FT* appears to be critical for FR sensitivity. The semi-sensitive accession (Hau-0) crossed to ColFRI(ft-1) had an F1 FR-flowering phenotype equivalent to Hau-0. This demonstrates that the FT allele in Hau-0 retains some FR-sensitive *FT* function; however, the F1 from Hau-0/ColFRI(ft-1) is later than ColFRI, indicating that the *FT* allele from Hau-0 is less capable of responding to FR compared to the *FT* allele from ColFRI (S5 Table). As expected due to *FT* dominance, F1 plants from crosses between the *ft* mutant and FR-sensitive accessions (Yo-0, HOG, and Nok-3) had a flowering phenotype equivalent to the FR-sensitive accession (S5 Table). Two semi-sensitive accessions (Et-0 and Sue-0) with moderately late FR-flowering times (43 and 48 RLN, respectively) flowered later when crossed to the *CO* mutant (56 and 58.5 RLN, respectively,) likely due to the dosage-dependent behavior of *CO*, and suggesting a functional *CO* in these accessions. Et-0 (semi-sensitive) crossed to FR-sensitive (sensitive) accessions (HOG and Nok-3) had flowering times equivalent to the FR-sensitive accessions, suggesting that the Et-0 *CO* allele is capable of acting on the *FT* alleles from those two accessions. In addition, the Et-0/ColFRI F2 showed no linkage at chromosome 5, demonstrating *CO* functionality in this accession (S5 Table).

The only accession with an amino acid substitution in *FT*, Fl-1, flowers extremely late in FR-enriched conditions (>80 RLN). The Fl-1/ColFRI(*ft-1*) F1 population flowers as late as Fl-1 and ColFRI(*ft-1*), demonstrating that the Fl-1 *FT* allele has an altered function. Fl-1/ColFRI(co) F1 flowering time data (54 RLN) shows that the Fl-1 *CO* allele appears functional and is capable of acting on the ColFRI *FT* allele, further supporting the notion that Fl-1 FR insensitivity of Fl-1 results exclusively from an altered functional *FT* allele. Therefore, the data support the notion that *FT* allelic variability in natural accessions is a major factor in reduced FR sensitivity for flowering time.

Using a Recombinant Inbred Lines (RIL) population, the Est-1 accession was shown to have a hypo-active *FT* allele, which mapped to the *FT* promoter region [32]. A NIL (Near-Isogenic Line) used to isolate the Est-1 *FT* region in a Col background was crossed to ColFRI to determine if this allele of *FT* was compromised in FR-dependent flowering in a winter-annual (FRI+) background. The FR-flowering phenotype of the F2 population was dependent on the *FT* region with plants containing Col *FT* (14 RLN) flowering substantially earlier than plants containing Est-1 *FT* (38.6 RLN), providing additional evidence that *FT* plays a central role in FR-induced flowering.

### *FT* Expression

**Fig 6:**
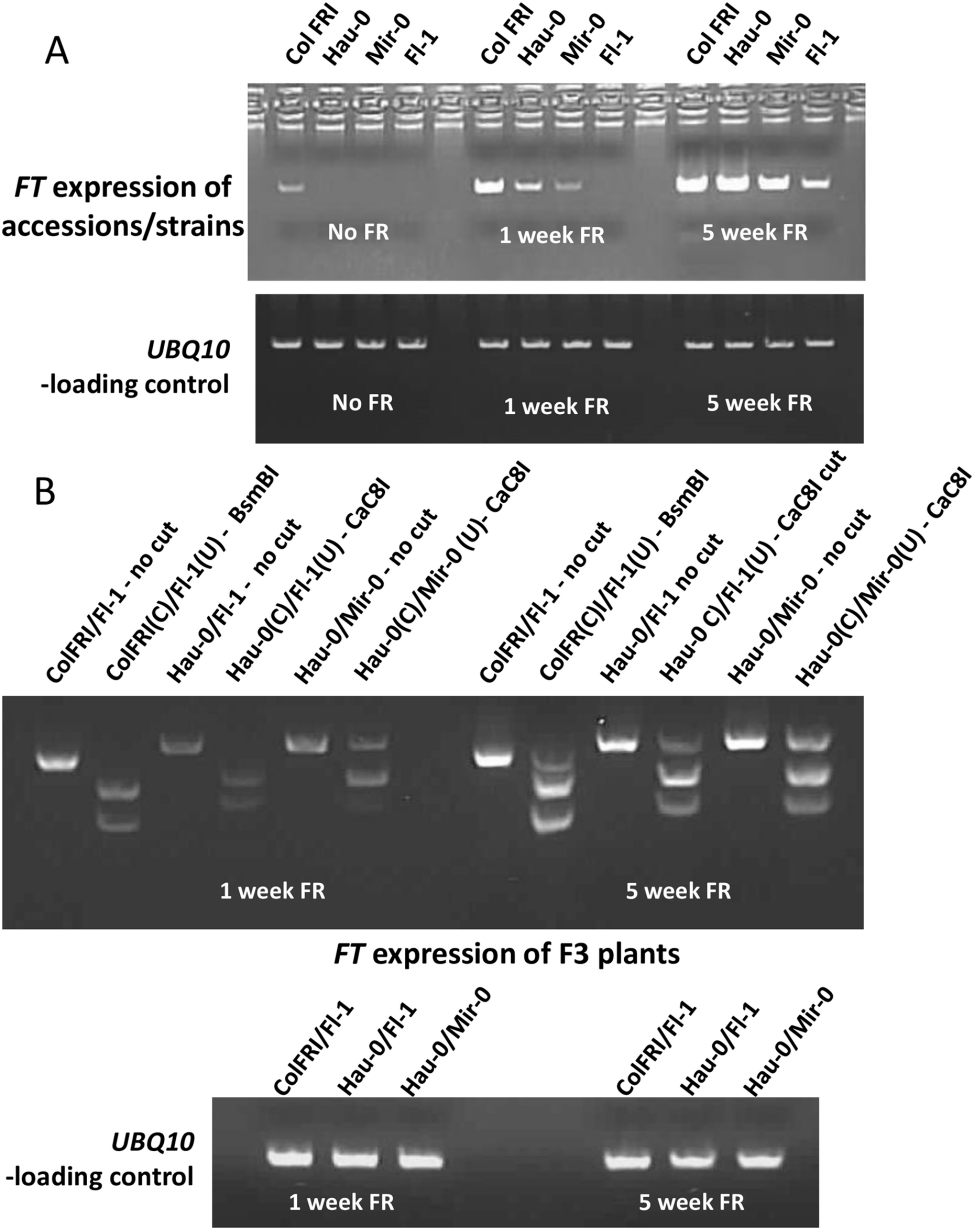
In multiple isogenic lines, *FT* expression is linked to FR sensitivity in an allele specific manner. A) Three accessions (Hau-0, Mir-0 and Fl-1) and the laboratory strain ColFRI were evaluated for *FT* expression. Plants were grown in LD conditions for 4 weeks and then moved to LD or LD + FR. Samples were collected 1 week and 5 weeks after being moved. B) Allele specific *FT* expression in F3 plants heterozygous for *FT*. BsmBI cuts the ColFRI and Hau-0 allele, whereas Cac8I cuts the Fl-1 allele. In the figure for allele specific *FT* expression, (C) = allele is cut with the enzyme, whereas (U) = allele is not cut by that specific enzyme.

We evaluated *FT* expression in 3 of our well-characterized accessions compromised in FR-enriched flowering and ColFRI, +/- FR (Fig 6A). Without FR addition, the only accession/strain that flowered was ColFRI, and *FT* expression is only detected in that background. After 1 week in FR-enriched conditions, *FT* expression is observed in ColFRI, Hau-0, and Mir-0, but not Fl-1, which is consistent with their flowering times under FR conditions (15RLN, 32RLN, 50RLN, and DNF, respectively). After 5 weeks of FR treatment, all accessions/strains were expressing FT with ColFRI and Hau-0 having relatively high and equivalent expression, which correlates well with the FR responsiveness. Both Mir-0 and Fl-1 had not flowered after 5 weeks of FR and this correlates with their lower level of *FT* expression.

To explore whether or not the *FT* alleles would be expressed at different levels, we evaluated F3 plants that are heterozygous at *FT* from 3 populations for allele-specific expression (Fig 6B). As expected from the above results, *FT* expression correlated with flowering time with ColFRI alleles having the highest/earliest expression followed by Hau-0, Mir-0, and Fl-1 alleles, respectively. These results demonstrate that in an identical genetic background, the four different *FT* alleles are differentially expressed under FR conditions.

## Discussion

**Fig 7:**
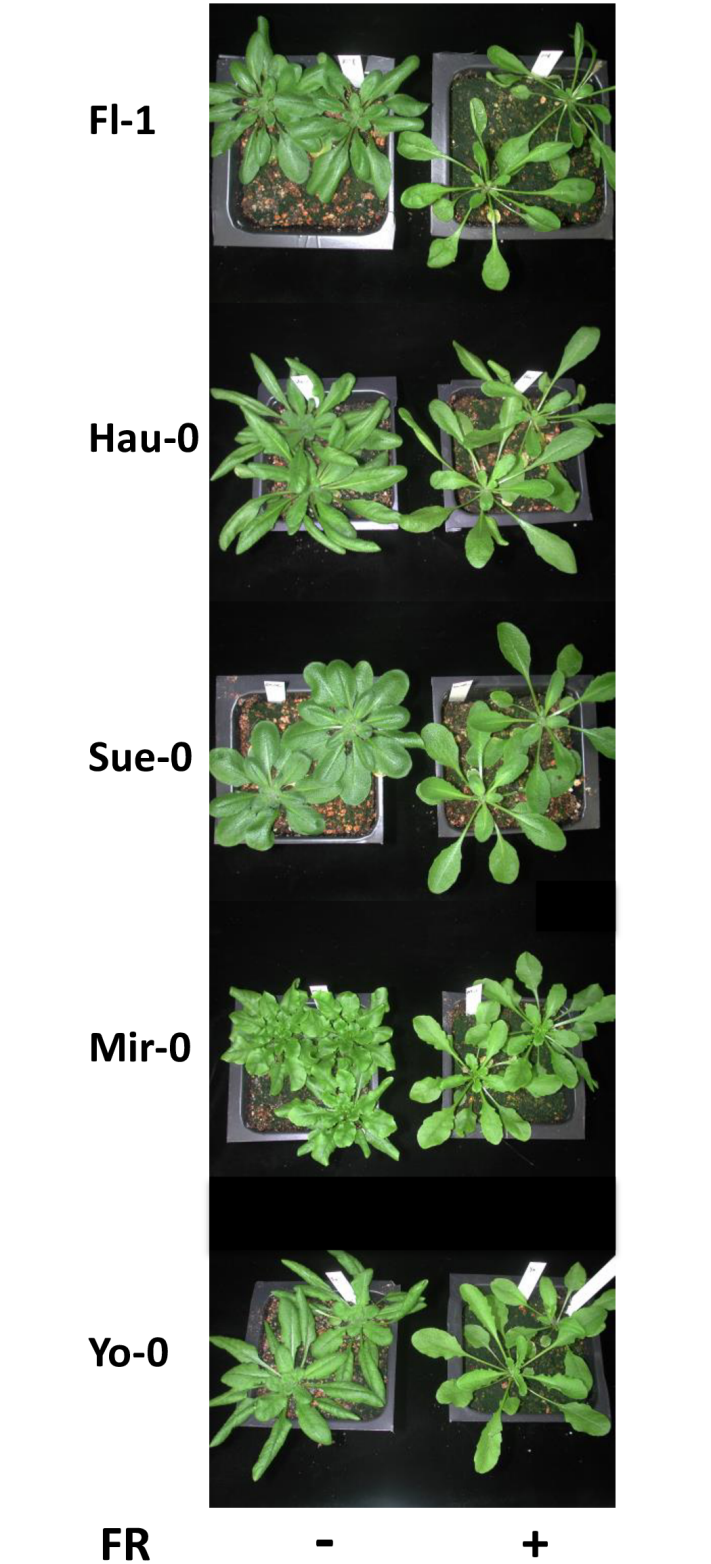
Petiole phenotypes, a classic FR enrichment response, are independent of flowering phenotypes. While having variable FR induced flowering phenotypes, all accessions tested display petiole elongation.

We identified a number of accessions that had an attenuated flowering response to FR. The genetic pathway promoting flowering under FR-enriched conditions is likely to be different than the pathway that influences hypocotyl elongation, because comparing FR and GA sensitivity for hypocotyl elongation from Maloof et al. [35] to accessions which are insensitive to FR-induced flowering showed no correlation (S4 Table). In addition, FR-dependent petiole elongation occurs in accessions that are sensitive, semi-sensitive and insensitive for FR-induced flowering (Fig 7). Thus, phenotypic responses to FR or shade likely utilize different downstream pathways to elicit different responses. A similar conclusion was reached by evaluating flowering time and petiole elongation in phytochrome mutants [36]. Whereas petiole elongation in shaded conditions may be more advantageous regardless of location at the beginning of the plants life cycle, precocious flowering could have deleterious effects on reproductive success.

An interesting aspect of this study is that the phenotype in laboratory FR-enriched conditions mirrored the phenotype of plants grown outside in a partially shaded site. However, the R:FR ratio in these two situations was substantially different. In the laboratory, we utilized two levels of FR enrichment, with R:FR ratios of 0.04 and 0.16. As measured by a spectroradiometer, the R:FR ratio outside was never below 0.60; however, overall light intensity was 3X higher. Thus, it is possible that at higher intensities, a higher R:FR ratio is effective in promoting flowering. Another possibility is that temperature cycling outdoors acts synergistically with FR to promote flowering. We did not, however, observe an effect of temperature cycling in this work, but it may have been masked by the effect of the low R:FR ratio we used in the laboratory.

Most accessions that do not show a rapid acceleration of flowering under FR-enriched conditions are from northern Europe (UK, Finland, Sweden, and Denmark). It is tempting to speculate that this behavior is not just a consequence of population structure, but provides an adaptive advantage to accessions at higher latitudes that experience a long winter. One possibility is that because northern latitudes experience longer durations of twilight which is enriched in FR, insensitivity to FR could prevent flowering at inappropriate times of the growing season [37, 38]. Another possibility is simply genetic drift; because these accessions reproducibly encounter cold yearly, there would be little selective pressure to maintain an additional pathway (photoperiod) to promote flowering.

Two accessions from southern Europe, Mir-0 (Miramare, Italy, 45.7025 N, 13.7125E) and Br-0 (Brunn, Czech Republic 49.1166 N, 16.6083), were also FR insensitive for flowering. Both accessions are glabrous, have identical flowering behavior, and were collected 378 miles apart, indicating the possibility of relatedness. These two accessions are also unique in that both *CO* and *FT* allelic variation is likely to contribute to insensitivity to FR-induced flowering. Why FR insensitivity is present in accessions from these two regions is unknown, but the relatively short vernalization requirement shared by these two accessions may render the lack of a FR response ecologically relevant, providing an additional layer of protection to prevent precocious flowering.

In summary, we demonstrate that *FT* expression is intimately associated with FR-induced flowering. In Arabidopsis, *FT* is one of the most highly conserved proteins with only a single amino acid variation across the species; thus, variability associated with FT must reside in expression of the *FT* gene, as demonstrated in this study. *FT* has both negative regulators (*FLC*, *SVP*, and *TEMPRANILLO*) as well as positive regulators (*CO*, *GI*, and *SQUAMOSA PROMOTER BINDING PROTEIN-LIKE 3*), all of which bind to the promoter region or the first intron [7, 39, 40]. Phylogenetic shadowing has identified conserved blocks in the region upstream of *FT* that could serve as binding sites for regulatory proteins that control *FT* transcription [21, 41]. Future studies evaluating differences in proteins and their interactions with promoter motifs will be useful in understanding the cooperative or competitive interactions controlling *FT* expression. In addition to regulators and promoter motifs, a role for chromatin modification has been demonstrated providing an additional layer of regulation [21]. The unusual genomic spacing of *FT*, with no ORFs 7.5kb upstream and 9.5kb downstream, may be related to regulation by higher-order chromatin structure. Thus, *FT* regulation is complex as might be expected for an integrator that receives signals from multiple pathways (photoperiod, temperature, light quality, and hormone levels). We have shown that FR enrichments leads to accelerated flowering in some accessions via variability in *FT* expression. FT, or florigen, is central to environmentally induced flowering and an attractive candidate for engineering plants for a changing environment.

## Supporting information

S1 Fig Flowering times in SD mirrored LD flowering times with application of exogenous GA. GA accelerated flowering for all accessions except Fl-1. Fl-1 grew extensive secondary meristems, making scoring RLN inaccurate, thus the SD experiment was terminated after 60 days.
(TIF)

S2 Fig F1 and parental flowering time of accessions covering the spectrum of FR sensitivity in FR-enriched conditions. F1 populations had flowering times that mirror the flowering time of the most FR sensitive parent demonstrating dominance, and no F1 exceeded the flowering time of the less sensitive parent.
(TIF)

S3 Fig FR enriched flowering time distribution for an F2 population from a FR insensitive and a FR sensitive accession. The F2 population from a cross between Fl-1 (In) and ColFRI (Se) segregated in a 3:1 manner, suggesting one major-effect loci as conferring the phenotype in this population.
(TIF)

S1 Table Phenotypic variation for flowering time in natural *Arabidopsis* accessions. Flowering time data for *Arabidopsis* accessions used in this study, along with controls (Col and ColFRI), under various growth conditions.
(XLSX)

S2 Table Flowering time phenotypes in 10 accessions. Ten accessions, representing much of the breadth in flowering time behavior, were phenotyped under 10 various growth conditions.
(XLSX)

S3 Table Correlations of flowering times for 10 accessions in various growth conditions. Correlations among the ten conditions for the 10 accessions from SD S2 Table.
(XLSX)

S4 Table Correlation of hypocotyl elongation and FR flowering time. Results for accessions contained within this study and those used my Maloof, et. al, show no correlation between FR responsiveness for the two FR dependent phenotypes.
(XLSX)

S5 Table F1 and parental FR enriched flowering times. F1 plants flower most like the early flowering (FR-sensitive) accessions, demonstrating dominance.
(XLSX)

S6 Table Oligos and markers used in this study. Over 100 accessions were scored for multiple polymorphisms around FT, with two being within the ORF of FT, which was used for the allele-specific expression experiments (BsmBI and Cac8I). Only two accessions (Fl-1 and Bsch-2) contained the polymorphism responsible for the BsmBI site. In addition, the table shows data for an indel upstream of FT (1.2, 1.4, and 2.3kb; Warthmann personal communication). The 1.2kb version is relatively rare being present in only 6 accessions, including two (Et-0 and Br-0), accessions used extensively in this study.
(XLSX)

## References

[1] Smith H. Light Quality, Photoperception, and Plant Strategy. Annual Review of Plant Physiology. 1982;33(1):481–518. doi: 10.1146/annurev.pp.33.060182.002405

[2] Frankilin KA, Whitelam, G. C., Shinkle, J. R. Photomorphogenesis in natural light environments. http://wwwphotobiologyinfo/Franklin-NLEhtml. 2008.

[3] Franklin KA, Larner VS, Whitelam GC. The signal transducing photoreceptors of plants. Int J Dev Biol. 2005;49(5–6):653–64. doi: 10.1387/ijdb.051989kf 16096972 10.1387/ijdb.051989kf

[4] Franklin KA, Praekelt U, Stoddart WM, Billingham OE, Halliday KJ, Whitelam GC. Phytochromes B, D, and E Act Redundantly to Control Multiple Physiological Responses in Arabidopsis. Plant Physiology. 2003;131(3):1340–6. doi: 10.1104/pp.102.015487 12644683 10.1104/pp.102.015487 PMC166893

[5] Filiault DL, Wessinger CA, Dinneny JR, Lutes J, Borevitz JO, Weigel D, et al Amino acid polymorphisms in Arabidopsis phytochrome B cause differential responses to light. PNAS. 2008;105(8):3157–62. doi: 10.1073/pnas.0712174105 18287016 10.1073/pnas.0712174105 PMC2268601

[6] Jiao Y, Lau OS, Deng XW. Light-regulated transcriptional networks in higher plants. Nat Rev Genet. 2007;8(3):217–30. doi: 10.1038/nrg2049 17304247 10.1038/nrg2049

[7] Sawa M, Kay SA. GIGANTEA directly activates Flowering Locus T in Arabidopsis thaliana. Proceedings of the National Academy of Sciences. 2011;108(28):11698–703. 10.1073/pnas.1106771108 PMC3136272 21709243

[8] Suarez-Lopez P, Wheatley K, Robson F, Onouchi H, Valverde F, Coupland G. CONSTANS mediates between the circadian clock and the control of flowering in Arabidopsis. Nature. 2001;410(6832):1116–20. doi: 10.1038/35074138 11323677 10.1038/35074138

[9] Yoo SK, Chung KS, Kim J, Lee JH, Hong SM, Yoo SJ, et al CONSTANS Activates SUPPRESSOR OF OVEREXPRESSION OF CONSTANS 1 through FLOWERING LOCUS T to Promote Flowering in Arabidopsis. Plant Physiology. 2005;139(2):770–8. doi: 10.1104/pp.105.066928 16183837 10.1104/pp.105.066928 PMC1255994

[10] Koornneef M, Hanhart CJ, Veen JH. A genetic and physiological analysis of late flowering mutants in Arabidopsis thaliana. Molec Gen Genet. 1991;229(1):57–66. doi: 10.1007/bf00264213 1896021 10.1007/BF00264213

[11] Kardailsky I, Shukla VK, Ahn JH, Dagenais N, Christensen SK, Nguyen JT, et al Activation Tagging of the Floral Inducer FT. Science. 1999;286(5446):1962–5. doi: 10.1126/science.286.5446.1962 10583961 10.1126/science.286.5446.1962

[12] Turnbull C. Long-distance regulation of flowering time. Journal of Experimental Botany. 2011;62(13):4399–413. doi: 10.1093/jxb/err191 21778182 10.1093/jxb/err191

[13] Navarro C, Abelenda JA, Cruz-Oro E, Cuellar CA, Tamaki S, Silva J, et al Control of flowering and storage organ formation in potato by FLOWERING LOCUS T. Nature. 2011;478(7367):119–22. Epub 2011/09/29. nature10431 [pii] doi: 10.1038/nature10431 . 21947007 10.1038/nature10431

[14] Böhlenius H, Huang T, Charbonnel-Campaa L, Brunner AM, Jansson S, Strauss SH, et al CO/FT Regulatory Module Controls Timing of Flowering and Seasonal Growth Cessation in Trees. Science. 2006;312(5776):1040–3. 16675663 10.1126/science.1126038

[15] Fan C, Hu R, Zhang X, Wang X, Zhang W, Zhang Q, et al Conserved CO-FT regulons contribute to the photoperiod flowering control in soybean. BMC Plant Biology. 2014;14(1%@ 1471–2229):9 doi: 10.1186/1471-2229-14-9 24397545 10.1186/1471-2229-14-9 PMC3890618

[16] Blazquez MA, Ahn JH, Weigel D. A thermosensory pathway controlling flowering time in Arabidopsis thaliana. Nat Genet. 2003;33(2):168–71. http://www.nature.com/ng/journal/v33/n2/suppinfo/ng1085_S1.html. 12548286 10.1038/ng1085

[17] Amasino R. Seasonal and developmental timing of flowering. The Plant Journal. 2010;61(6):1001–13. doi: 10.1111/j.1365-313X.2010.04148.x 20409274 10.1111/j.1365-313X.2010.04148.x

[18] Domagalska MA, Sarnowska E, Nagy F, Davis SJ. Genetic Analyses of Interactions among Gibberellin, Abscisic Acid, and Brassinosteroids in the Control of Flowering Time in Arabidopsis thaliana. PLoS ONE. 2010;5(11):e14012 doi: 10.1371/journal.pone.0014012 21103336 10.1371/journal.pone.0014012 PMC2984439

[19] Lee JH, Yoo SJ, Park SH, Hwang I, Lee JS, Ahn JH. Role of SVP in the control of flowering time by ambient temperature in Arabidopsis. Genes Dev. 2007;15:397–402. 10.1101/gad.1518407 PMC1804328 17322399

[20] Strange A, Li P, Lister C, Anderson J, Warthmann N, Shindo C, et al Major-effect alleles at relatively few loci underlie distinct vernalization and flowering variation in Arabidopsis accessions. PLoS One. 2011;6(5):e19949 Epub 2011/06/01. doi: 10.1371/journal.pone.0019949 21625501 10.1371/journal.pone.0019949 PMC3098857

[21] Adrian J, Farrona S, Reimer JJ, Albani MC, Coupland G, Turck F. cis-Regulatory Elements and Chromatin State Coordinately Control Temporal and Spatial Expression of FLOWERING LOCUS T in Arabidopsis. The Plant Cell Online. 2010;22(5):1425–40. 10.1105/tpc.110.074682 PMC2899882 20472817

[22] Blackman BK, Strasburg JL, Raduski AR, Michaels SD, Rieseberg LH. The role of recently derived FT paralogs in sunflower domestication. Curr Biol. 2010;20(7):629–35. Epub 2010/03/23. doi: 10.1016/j.cub.2010.01.059 20303265 10.1016/j.cub.2010.01.059 PMC2898918

[23] Searle I, He Y, Turck F, Vincent C, Fornara F, Kr√∂ber S, et al The transcription factor FLC confers a flowering response to vernalization by repressing meristem competence and systemic signaling in Arabidopsis. Genes & Development. 2006;20(7):898–912. 16600915 10.1101/gad.373506 PMC1472290

[24] Li D, Liu C, Shen L, Wu Y, Chen H, Robertson M, et al A repressor complex governs the integration of flowering signals in Arabidopsis. Dev Cell. 2008;15:110–20. doi: 10.1016/j.devcel.2008.05.002 18606145 10.1016/j.devcel.2008.05.002

[25] Porri A, Torti S, Romera-Branchat M, Coupland G. Spatially distinct regulatory roles for gibberellins in the promotion of flowering of Arabidopsis under long photoperiods. Development. 2010;139:2190–209. 10.1242/dev.077164 22573618

[26] Wollenberg AC, Strasser B, Cerdan PD, Amasino RM. Acceleration of flowering during shade avoidance in Arabidopsis alters the balance between FLOWERING LOCUS C-mediated repression and photoperiodic induction of flowering. Plant Physiology. 2008;148:1681–94. doi: 10.1104/pp.108.125468 18790998 10.1104/pp.108.125468 PMC2577263

[27] Adams S, Allen T, Whitelam GC. Interaction between the light quality and flowering time pathways in Arabidopsis. The Plant Journal. 2009;60(2):257–67. doi: 10.1111/j.1365-313X.2009.03962.x 19563438 10.1111/j.1365-313X.2009.03962.x

[28] Rantanen M, Kurokura T, Mouhu K, Pinho P, Tetri E, Halonen L, et al Light quality regulates flowering in FvFT1/FvTFL1 dependent manner in the woodland strawberry Fragaria vesca. Frontiers in Plant Science. 2014;5:271 doi: 10.3389/fpls.2014.00271 24966865 10.3389/fpls.2014.00271 PMC4052200

[29] Kim SY, Yu X, Michaels SD. Regulation of CONSTANS & FLOWERING LOCUS T Expression in Response to Changing Light Quality. Plant Physiology. 2008;148(1):269 doi: 10.1104/pp.108.122606 18667727 10.1104/pp.108.122606 PMC2528114

[30] Lee I, Michaels SD, Masshardt AS, Amasino RM. The late-flowering phenotype of FRIGIDA and mutations in LUMINIDEPENDENS is suppressed in the Landsberg erecta strain of Arabidopsis. The Plant Journal. 1994;6(6):903–9. doi: 10.1046/j.1365-313X.1994.6060903.x

[31] Balasubramanian S, Sureshkumar S, Lempe J, Weigel D. Potent Induction of Arabidopsis thaliana Flowering by Elevated Growth Temperature. PLoS Genet. 2006;2(7):e106 doi: 10.1371/journal.pgen.0020106 16839183 10.1371/journal.pgen.0020106 PMC1487179

[32] Schwartz C, Balasubramanian S, Warthmann N, Michael TP, Lempe J, Sureshkumar S, et al Cis-regulatory Changes at FLOWERING LOCUS T Mediate Natural Variation in Flowering Responses of Arabidopsis thaliana. Genetics. 2009;183(2):723 doi: 10.1534/genetics.109.104984 19652183 10.1534/genetics.109.104984 PMC2766330

[33] Lee J, Yun JY, Zhao W, Shen WH, Amasino RM. A methyltransferase required for proper timing of the vernalization response in Arabidopsis. Proc Natl Acad Sci U S A. 2015;112(7):2269–74. Epub 2015/01/22. doi: 10.1073/pnas.1423585112 25605879 10.1073/pnas.1423585112 PMC4343098

[34] Warthmann N, Fitz J, Weigel D. MSQT for choosing SNP assays from multiple DNA alignments. Bioinformatics. 2007;23(20):2784–7. doi: 10.1093/bioinformatics/btm428 17785349 10.1093/bioinformatics/btm428

[35] Maloof JN, Borevitz JO, Weigel D, Chory J. Natural variation in phytochrome signaling. Seminars in Cell & Developmental Biology. 2000;11(6):523–30. http://dx.doi.org/10.1006/scdb.2000.0198. 11145882 10.1006/scdb.2000.0198

[36] Halliday KJ, Salter MG, Thingnaes E, Whitelam GC. Phytochrome control of flowering is temperature sensitive and correlates with expression of the floral integrator FT. The Plant Journal. 2003;33(5):875–85. doi: 10.1046/j.1365-313X.2003.01674.x 12609029 10.1046/j.1365-313x.2003.01674.x

[37] Casal JJ, Sanchez RA, Gibson G. The significance of changes in the red/farred ratio, associated with either neighbour plants or twihght, for tillering in Lolium multiflorum Lam. New Pytol. 1990;116:565–72.

[38] Linkosalo T, Lechowicz MJ. Twilight far-red treatment advances leaf bud burst of silver birch (Betula pendula). Tree Physiol. 2006;26:1249–56. 16815827 10.1093/treephys/26.10.1249

[39] Osnato M, Castillejo C, Matias-Hernandez L, Pelaz S. TEMPRANILLO genes link photoperiod and gibberellin pathways to control flowering in Arabidopsis. Nat Commun. 2012;3:808 http://www.nature.com/ncomms/journal/v3/n5/suppinfo/ncomms1810_S1.html. doi: 10.1038/ncomms1810 22549837 10.1038/ncomms1810

[40] Kim JJ, Lee JH, Kim W, Jung HS, Huijser P, Ahn JH. The microRNA156-SQUAMOSA PROMOTER BINDING PROTEIN-LIKE3 Module Regulates Ambient Temperature-Responsive Flowering via FLOWERING LOCUS T in Arabidopsis. Plant Physiology. 2012;159(1):461–78. doi: 10.1104/pp.111.192369 22427344 10.1104/pp.111.192369 PMC3375978

[41] Wang J, Hopkins CJ, Hou J, Zou X, Wang C, Long Y, et al Promoter Variation and Transcript Divergence in Brassicaceae Lineages of FLOWERING LOCUS T. PLoS ONE. 2012;7(10):e47127 doi: 10.1371/journal.pone.0047127 23071733 10.1371/journal.pone.0047127 PMC3469537

