# Supplementary figures and images for: Variation in shade-induced flowering in Arabidopsis thaliana results from FLOWERING LOCUS T allelic variation
Source: PLoS One. 2017 Nov 8;12(11):e0187768. doi: 10.1371/journal.pone.0187768 (PMC5695581; doi:10.1371/journal.pone.0187768)

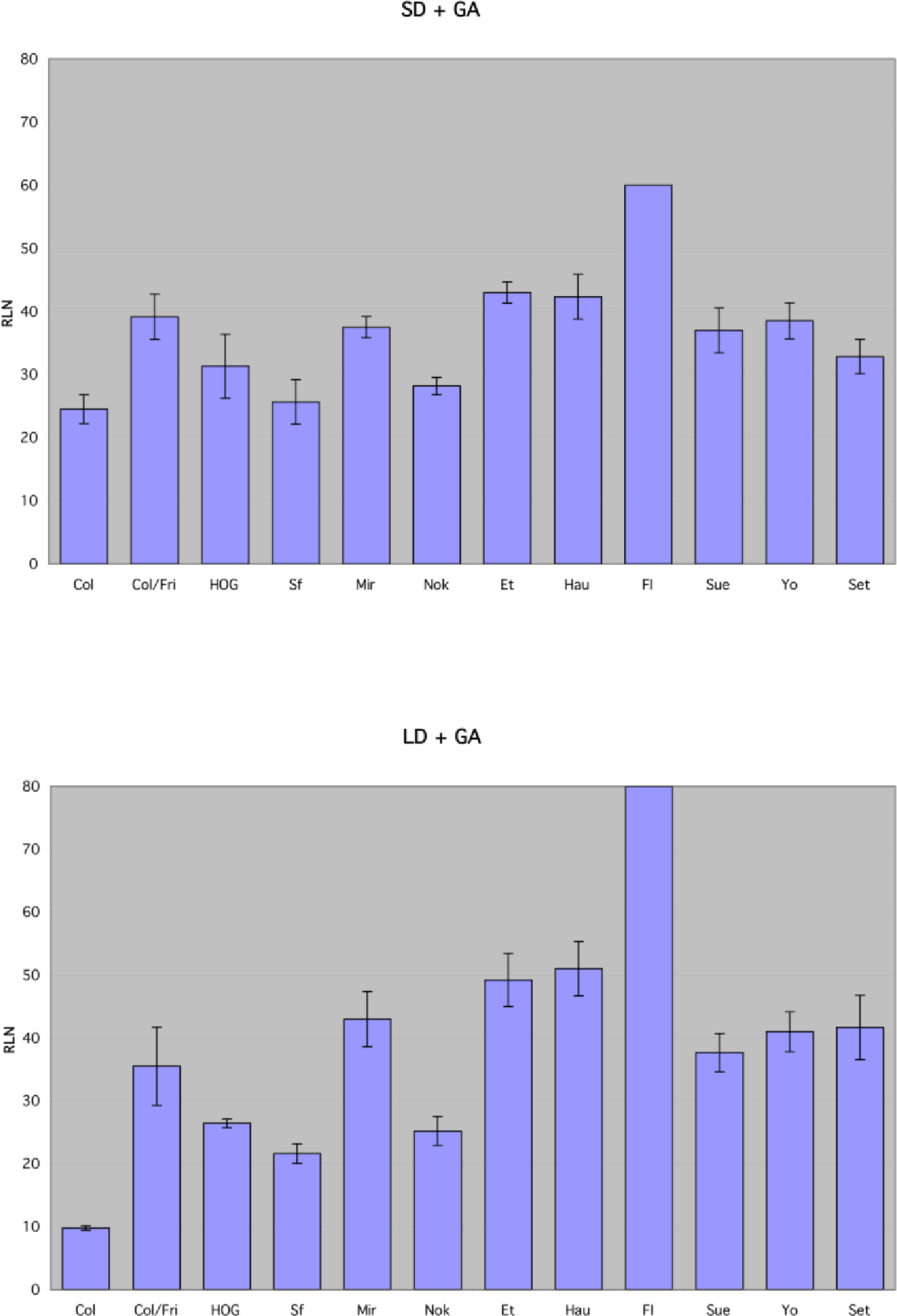

Supplement: S1 Fig — GA accelerated flowering for all accessions except Fl-1. Fl-1 grew extensive secondary meristems, making scoring RLN inaccurate, thus the SD experiment was terminated after 60 days. (TIF) [file pone.0187768.s001.tif]

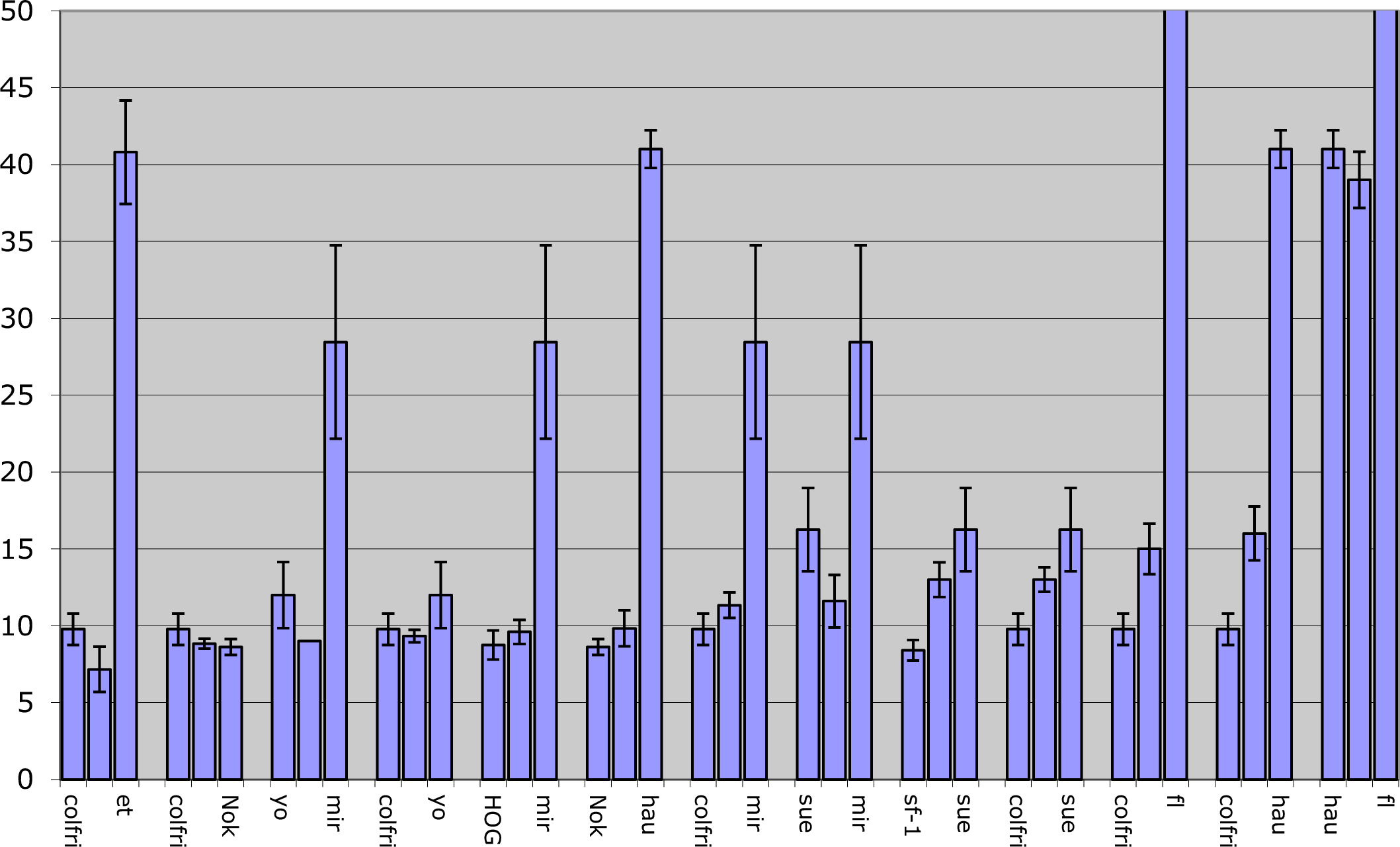

Supplement: S2 Fig — F1 populations had flowering times that mirror the flowering time of the most FR sensitive parent demonstrating dominance, and no F1 exceeded the flowering time of the less sensitive parent. (TIF) [file pone.0187768.s002.tif]

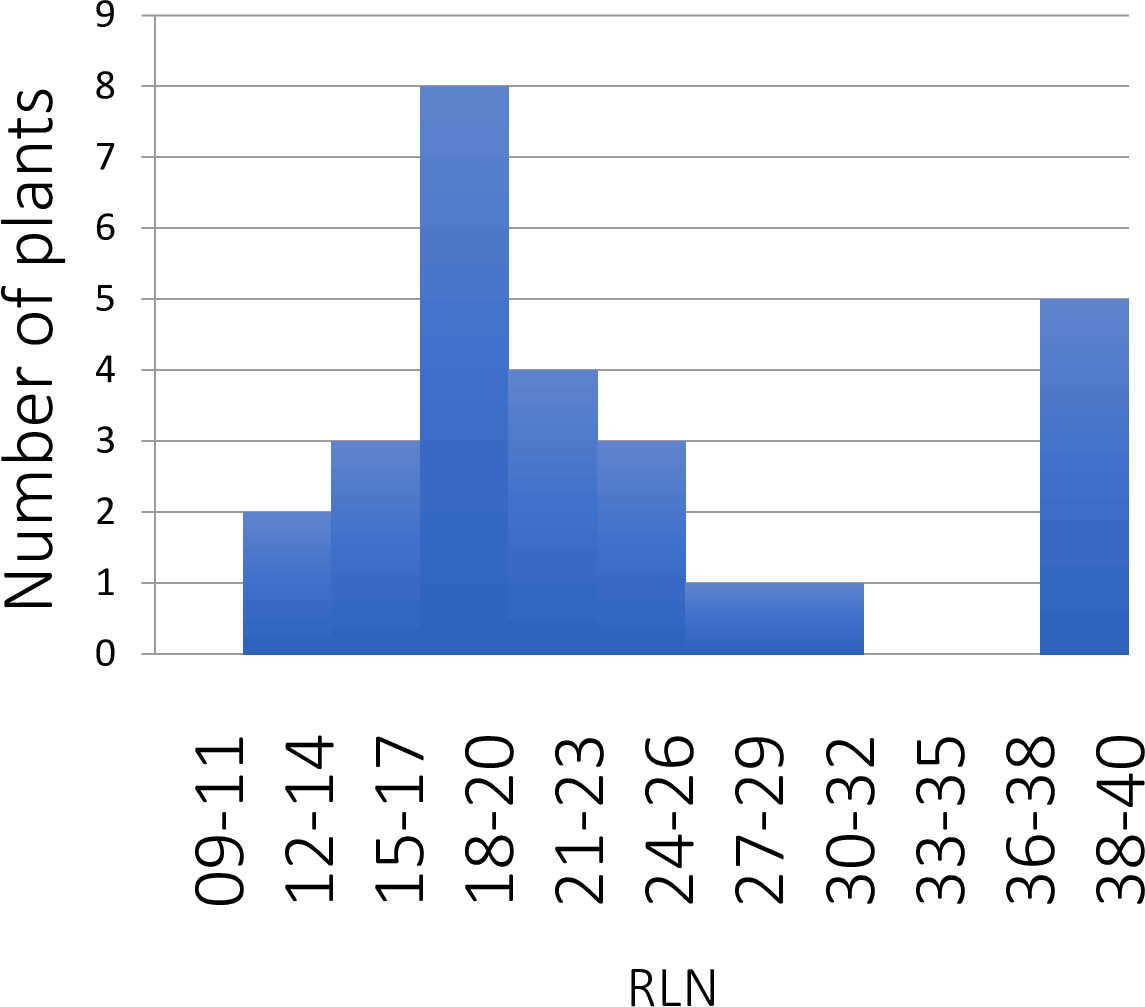

Supplement: S3 Fig — The F2 population from a cross between Fl-1 (In) and ColFRI (Se) segregated in a 3:1 manner, suggesting one major-effect loci as conferring the phenotype in this population. (TIF) [file pone.0187768.s003.tif]
